# Genomic prediction in a backcross population using relationship matrices

**DOI:** 10.5194/aab-68-377-2025

**Published:** 2025-06-17

**Authors:** Abdulraheem A. Musa, Jan Klosa, Manfred Mayer, Norbert Reinsch

**Affiliations:** 1 Research Institute for Farm Animal Biology (FBN), Wilhelm-Stahl-Allee 2, 18196 Dummerstorf, Germany; 2 Department of Animal Production, Kogi State University, Anyigba, Nigeria

## Abstract

Backcrossing is a widely used crossbreeding strategy for transferring desirable traits from a donor line into a recurrent parent population. Although genomic selection can accelerate genetic improvement in these populations, traditional models such as G-BLUP (genomic best linear unbiased prediction) often assume marker independence and uniform variance contributions – simplifications that can affect the accuracy of genomic estimated breeding values (GEBVs). To address these shortcomings, we developed three models: covariance-adjusted genomic BLUP (CAG-BLUP), which accounts for correlated markers, and two genomic-architecture-specific BLUP variants (GASI-BLUP for independent markers and GASC-BLUP for correlated markers), both assuming unequal variance contributions. Evaluated against the conventional G-BLUP using simulated and empirical mouse datasets, these models demonstrated superior performance in predicting GEBVs and in capturing genetic-architecture nuances. Specifically, GASI-BLUP significantly outperformed G-BLUP in scenarios involving independent quantitative trait loci (QTLs), improving GEBV prediction accuracy by up to 12 % and reducing underestimation of genetic variance by 12 %–34 %. CAG-BLUP showed enhanced performance in dependent QTL scenarios, particularly at lower heritabilities, with an improvement of up to 2 % in GEBV accuracy. These findings highlight the importance of selecting genomic prediction models tailored to the specific genetic architecture of traits to enhance prediction accuracy. By doing so, we pave the way for more effective breeding programs, promising substantial genetic improvements and contributing to the overarching goal of bolstering global food security. Future research will focus on expanding these models to incorporate non-additive genetic effects and on testing their applicability across different species and breeding contexts, aiming to further refine genomic prediction methodologies.

## 1 Introduction

Crossbreeding programs, supported by advanced genomic selection (GS) methodologies, are at the forefront of modern plant and animal breeding. By harnessing genetic variation across populations, breeders can systematically enhance economically important traits – such as productivity, adaptability, and health. Among various crossbreeding approaches, backcrossing has been particularly successful for transferring favorable traits from a donor line into a recurrent parent population (Hospital, 2005). However, such structured breeding often results in populations with complex linkage disequilibrium (LD) patterns, which affect the accurate prediction of true breeding values (TBVs). Because TBVs are influenced by the combined effects of multiple quantitative trait loci (QTLs) (Lynch and Walsh, 2018), tackling these complexities requires building upon foundational GS methodological differences, such as those introduced by Meuwissen et al. (2001), to develop more robust predictive models.

In GS, models are classified into direct and indirect methods depending on their utilization of genetic markers (Gao et al., 2015). Genomic best linear unbiased prediction (G-BLUP), a direct method, directly estimates genomic estimated breeding values (GEBVs) by integrating a genomic relationship matrix (GRM) derived from single nucleotide polymorphism (SNP) markers into mixed model equations (Hayes et al., 2009). In contrast, SNP-BLUP, an indirect method, estimates the effects of all SNP markers and aggregates them to calculate GEBVs (Meuwissen et al., 2001). Despite their methodological differences, both G-BLUP and SNP-BLUP are equivalent (Goddard, 2009; Hayes et al., 2009; Strandén and Garrick, 2009) in the sense indicated by Henderson (1985); that is, the expectation and variance in the observations are identical. This equivalence facilitates computational efficiency, enabling breeders to handle large-scale genomic datasets effectively.

Genetic variance is a cornerstone of heritability estimation and of predicting genetic gain (Falconer and Mackay, 1996). However, genomic prediction models that assume marker independence and uniform variance contributions often bias genetic variance estimates. Hayes and Goddard (2001) highlight the fact that quantitative traits are typically influenced by loci with varying effect sizes, challenging the assumption that all loci contribute equally. QTL mapping studies, such as those by Mackay (2001), further reveal that most quantitative traits are controlled by a finite number of loci, underscoring the importance of accounting for unequal marker contributions. Consequently, such models often over-shrink large marker effects toward the mean, reducing the accuracy of genomic predictions (Habier et al., 2007). Addressing these limitations requires optimizing shrinkage parameters, which depend on factors such as the trait's genetic architecture, heritability, and number of markers and the amount of LD in the data (Kärkkäinen and Sillanpää, 2012).

In recent years, there has been an increasing interest in utilizing covariance or correlation structures in GS to account for LD between markers (Mathew et al., 2017; Speed et al., 2012). This is particularly crucial in populations where LD is known to be high, such as early-segregating generations like F2 or backcross populations, mainly due to the physical linkage of loci (Hill et al., 2008; Tanksley, 1993). Notably, Mathew et al. (2017) made a significant contribution to this field by utilizing LD patterns to improve the derivation of GRMs. However, this approach has certain limitations, including reliance on an iterative process and cross-validation for LD decay estimation, as well as a high computational burden for calculating pairwise LD and inverting the LD matrix.

In addressing the existing limitations of GS in backcross populations, our study introduces the covariance-adjusted genomic BLUP (CAG-BLUP) model. This model incorporates a covariance matrix developed by Bonk et al. (2016) for full sibs to account for marker correlations resulting from LD. Furthermore, we present the newly developed genomic-architecture-specific BLUP (GAS-BLUP) strategy and its two variants: GAS-BLUP with independent markers (GASI-BLUP) and GAS-BLUP with correlated markers (GASC-BLUP). Contrarily to using a whole-genome shrinkage parameter, a chromosome-specific shrinkage parameter, in theory, would be more optimal but computationally prohibitive. As an intermediate solution, GAS-BLUP employs two shrinkage parameters aimed at genome segments with varying significance for QTLs, thus achieving a more refined representation of the genetic architecture of traits while enhancing prediction accuracy in a computationally efficient manner.

In this study, we evaluate these novel models using a first-generation backcross (BC1) population derived from two inbred lines, an ideal system for examining the effects of LD and uneven marker variance. Specifically, we introduce and compare three models: (1) CAG-BLUP, which leverages a covariance matrix to reflect marker correlations; (2) GASI-BLUP, assuming independent markers; and (3) GASC-BLUP, considering correlated markers. All GAS-BLUP variants assume an unequal contribution of markers to genetic variance. Additionally, we introduce their equivalent linear models. Our objective is to discern the properties of these models and to compare their performance with the G-BLUP model based on three key metrics: the precision of additive genetic variability estimates; the accuracy of GEBVs; and the predictive ability under diverse trait genetic architectures, heritabilities, and marker densities. By providing a thorough analysis of these models, we aim to contribute to the design of more effective breeding schemes and, ultimately, to enhance genetic gain in backcross programs.

## 2 Materials and methods

### 2.1 Derivation of marker-derived genomic relationship matrices

This section outlines the methodology employed for deriving GRMs, which is essential for estimating the GEBVs of genotyped individuals. The GEBVs for genotyped individuals ( n ) can be estimated using a GRM and BLUP. A GRM assuming that all markers are independent and contribute equal variance is equivalent to the matrix G in G-BLUP (VanRaden, 2008). For backcross populations, this matrix can be written as follows:

1 $$G=\left({\mathrm{ZIZ}}^{\prime }\right)\cdot \frac{1}{p},$$

where p is the number of markers (parameters), I is an n×n identity matrix indicating the independence of markers, and Z is a matrix containing genotype codes. We code genotypes in Z as follows:

$$z_{ij}=\left\{\begin{array}{ll} +1, & \text{for homozygous genotypes}\\ -1, & \text{for heterozygous genotypes} \end{array}\right.,$$

where i=1,…,n , and j=1,…,p . This coding reflects the unique structure of backcross populations involving inbred lines, where one parent contributes segregating alleles and the other provides fixed homozygous alleles.

Because BC populations with inbred parents can exhibit strong within-family LD between markers, we employ a covariance matrix R developed by Bonk et al. (2016) for full sibs. This matrix helps capture marker correlations stemming from LD. Specifically, we assume that inbred parental lines share identical genotypes, creating a consistent LD pattern in BC populations. Given these assumptions, a CAG relationship matrix $G_{\mathrm{CAG}}$ is defined as follows:

2 $$G_{\mathrm{CAG}}=\left({\mathrm{ZRZ}}^{\prime }\right)\cdot \frac{1}{s},s=1^{\prime }R1.$$

In this equation, R represents the covariance matrix, s is a scaling factor calculated as the sum of all elements in R , and 1 is a vector of ones. The element $r_{ij}$ of R for each chromosome is computed using Haldane's mapping function (Haldane, 1919), $r_{ij}=\mathrm{exp}\left(-2d_{ij}\right)$ , where $d_{ij}$ is the genetic distance between the two markers in morgans.

In a backcross population, the covariance matrix is equal to the correlation matrix because the variance in each marker locus satisfies $r_{ii}=\mathrm{exp}\left(0\right)=1$ . With a focus solely on additive effects, R is structured with one block per chromosome and exhibits an auto-regressive variance pattern, with unity along its diagonal. As identified in time series analysis (Seber, 2008), the inverse of R is known to be sparse and tridiagonal, allowing for direct setup from the genetic marker map and the distances between respective markers.

### 2.2 Linear models and their applications

#### 2.2.1 CAG-BLUP model and its equivalent linear model

For simplicity, we consider a linear model with a single fixed effect:

3 y=1μ+Wg+e.

Here, y is an n×1 vector of phenotypic values, 1 is an n -dimensional vector of ones, μ denotes the overall mean, W is an n×n design matrix relating records of GEBVs, g is an n×1 vector of GEBVs, and e is an n×1 vector of random residuals. We assume that the residuals are independent and normally distributed: $e∼N\left(0,I\sigma_{e}^{2}\right)$ , where $\sigma_{e}^{2}$ is the residual variance component. GEBVs are assumed to follow a normal distribution, $g∼N\left(0,G_{\mathrm{CAG}}\sigma_{a}^{2}\right)$ , differing from G-BLUP, which assumes $g∼N\left(0,G\sigma_{a}^{2}\right)$ , with $\sigma_{a}^{2}$ representing the additive genetic variance component.

The mixed model equations for Eq. (3) are represented as follows:

4 $$\left[\begin{array}{cc} 1^{\prime }1 & 1^{\prime }W\\ W^{\prime }1 & W^{\prime }W+G_{\mathrm{CAG}}^{-1}\cdot \sigma_{e}^{2}/\sigma_{a}^{2} \end{array}\right]\left[\begin{array}{c} \hat{\mu }\\ \hat{g} \end{array}\right]=\left[\begin{array}{c} 1^{\prime }y\\ W^{\prime }y \end{array}\right].$$

We estimate variance components using the restricted maximum likelihood method. The marker effects $\hat{m}$ in the CAG-BLUP model are calculated as follows:

5 $$\hat{m}={\mathrm{RZ}}^{\prime }G_{\mathrm{CAG}}^{-1}\cdot \frac{1}{s}\cdot \hat{g}.$$

Refer to Appendix A for the CAG-BLUP's equivalent SNP-BLUP model and to Appendix B for the proof of equivalence.

#### 2.2.2 GAS-BLUP models and their equivalent linear models

The GAS-BLUP models are executed in two steps, tailored to identify and analyze QTL presence across chromosomes. Initially, G-BLUP is utilized to precede GASI-BLUP, and CAG-BLUP is specifically applied before GASC-BLUP, assessing QTLs on each chromosome. This stage classifies the genome into segments of significant or non-significant for QTLs, which are then analyzed using the corresponding GASI-BLUP or GASC-BLUP models.

QTL-carrying chromosomes are identified through a likelihood-ratio test, contrasting the log-likelihoods of G-BLUP or CAG-BLUP against a null model. Significance is determined at 1 degree of freedom with P values, adjusted for multiple comparisons using Bonferroni correction (Holm, 1979), and a threshold of P<0.05 denotes significance.

The model for GASC-BLUP is as follows:

6 $$y=1\mu +Wg_{\mathrm{sg}}+Wg_{\mathrm{nsg}}+e.$$

In this equation, $g_{\mathrm{sg}}$ and $g_{\mathrm{nsg}}$ represent GEBV vectors for significant and non-significant genome segments, respectively. The GASC-BLUP differs from GASI-BLUP in terms of its relationship matrix implementation. The mixed model equations for Eq. (6) are

7 $$\begin{array}{rl} & \left[\begin{array}{ccc} 1^{\prime }1 & 1^{\prime }W & 1^{\prime }W\\ W^{\prime }1 & W^{\prime }W+G_{{\mathrm{CAG}}_{\mathrm{sg}}}^{-1}\cdot \sigma_{e}^{2}/\sigma_{a_{\mathrm{sg}}}^{2} & W^{\prime }W\\ W^{\prime }1 & W^{\prime }W & W^{\prime }W+G_{{\mathrm{CAG}}_{\mathrm{nsg}}}^{-1}\cdot \sigma_{e}^{2}/\sigma_{a_{\mathrm{nsg}}}^{2} \end{array}\right]\\ & \left[\begin{array}{c} \mu \\ {\hat{g}}_{\mathrm{sg}}\\ {\hat{g}}_{\mathrm{nsg}} \end{array}\right]=\left[\begin{array}{c} 1^{\prime }y\\ W^{\prime }y\\ W^{\prime }y \end{array}\right]. \end{array}$$

Here, $\sigma_{a_{\mathrm{sg}}}^{2}$ and $\sigma_{a_{\mathrm{nsg}}}^{2}$ represent the additive genetic variance components for significant and non-significant genome segments. GEBVs are calculated as $\hat{g}={\hat{g}}_{\mathrm{sg}}+{\hat{g}}_{\mathrm{nsg}}$ , and the estimated marker effects are derived using

8 $$\begin{array}{l} {\hat{m}}_{\mathrm{sg}}=R_{\mathrm{sg}}{Z^{\prime }}_{\mathrm{sg}}G_{{\mathrm{CAG}}_{\mathrm{sg}}}^{-1}\cdot \frac{1}{s_{\mathrm{sg}}}\cdot {\hat{g}}_{\mathrm{sg}}\\ {\hat{m}}_{\mathrm{nsg}}=R_{\mathrm{nsg}}{Z^{\prime }}_{\mathrm{nsg}}G_{{\mathrm{CAG}}_{\mathrm{nsg}}}^{-1}\cdot \frac{1}{s_{\mathrm{nsg}}}\cdot {\hat{g}}_{\mathrm{nsg}} \end{array}.$$

For the GASI-BLUP model, which employs the original GRM G instead of the $G_{\mathrm{CAG}}$ , the estimated marker effects are calculated using

9 $$\begin{array}{l} {\hat{m}}_{\mathrm{sg}}={Z^{\prime }}_{\mathrm{sg}}G_{\mathrm{sg}}^{-1}\cdot \frac{1}{p_{\mathrm{sg}}}\cdot {\hat{g}}_{\mathrm{sg}}\\ {\hat{m}}_{\mathrm{nsg}}={Z^{\prime }}_{\mathrm{nsg}}G_{\mathrm{nsg}}^{-1}\cdot \frac{1}{p_{\mathrm{nsg}}}\cdot {\hat{g}}_{\mathrm{nsg}} \end{array}.$$

Refer to Appendix C for the GAS-BLUP equivalent linear marker models and to Appendix D for the proof of equivalence.

### 2.3 Simulated dataset

We conducted two sets of simulations, categorized as independent and dependent simulations. These simulations were implemented using a custom-written Fortran 95 program, providing precise control over genetic parameters and environmental effects.

In the independent simulation, we modeled a BC1 population derived by crossing F1 individuals (from two inbred parental lines) with the recurrent parent. This design creates a unique genetic structure characterized by high LD across the genome, driven by limited recombination events and fixed parental alleles. Such LD patterns resemble those found in structured populations like full-sib families, justifying the use of the covariance matrix tailored to full-sib relationships (Bonk et al., 2016).

The simulated population consisted of 100 000 individuals, each possessing 20 chromosomes, reflecting the mouse genome structure. The chromosomes were each 100 cM (centimorgans) long. To mimic a high-density genotyping environment typical in mouse studies, we evenly distributed 101 markers on each chromosome, resulting in a marker density of 2020 markers per genome. Recombination rates between these markers were determined using Haldane's mapping function (Haldane, 1919). Subsequently, we introduced 12 QTLs with effect sizes ranging from - 0.25 to 1.00 at exact marker locations on the first 12 chromosomes. The cumulative effect of these QTLs constituted the TBV for each individual. Phenotypic values were derived by adding random residual effects to the TBV.

To assess the model's performance across different genetic architectures, we simulated three heritability scenarios: low ( $h^{2}=0.17$ ), medium ( $h^{2}=0.29$ ), and high ( $h^{2}=0.70$ ). Additionally, we adjusted the marker density by removing markers, resulting in densities of 420 and 220 markers on the genome, spaced at 5 and 10 cM intervals, respectively (Table 1). In these scenarios, QTLs were located at exact marker locations and between-marker positions. In each simulation scenario, we divided the 100 000 individuals into 200 replicates, each consisting of 500 individuals. This division was implemented to explore different relationships between the number of parameters ( p ) and the number of individuals ( n ). Specifically, we examined situations where p≫n , p≈n , and p<n , corresponding to marker densities of 2020, 420, and 220 markers, respectively.

**Table 1:** QTL positions and their effect sizes in independent and dependent QTL simulations of backcross populations.

	Marker density			
Independent simulation	220	420	2020	Effect size
Chromosome				
1	6	11	51	0.50
2	4–5	7–8	34	1.00
3	7–8	14–15	67	0.25
4	1–2	2	6	0.50
5	10–11	20	96	0.50
6	4–5	8–9	40	0.10
7	6–7	12–13	60	0.25
8	6	11	51	0.25
9	4–5	7–8	34	- 0.25
10	7–8	14–15	67	0.50
11	3	5	21	1.00
12	9	17	81	0.10
Dependent simulation				
Chromosome				
1	3	5	21	1.00
	4–5	7–8	34	1.00
	10–11	20	96	0.50
2	1–2	2	6	0.50
	6	11	51	0.50
	6–7	12–13	60	0.25
	7–8	14–15	67	0.50
3	4–5	8–9	40	0.10
	7–8	14–15	67	0.25
	9	17	81	0.10
4	4–5	7–8	34	- 0.25
	6	11	51	0.25

Independent simulations feature a single quantitative trait locus (QTL) per chromosome for the first 12 chromosomes, whereas dependent simulations include multiple QTLs when coupling on the first three chromosomes and two QTLs with repulsion on the fourth. QTLs are positioned at precise marker locations or in between two markers. The simulations cover marker densities of 220, 420, and 2020 under heritability scenarios of 0.17, 0.29, and 0.70.

The dependent simulation mirrored the independent simulation in terms of heritability and marker density. However, it featured a more intricate genetic setup, with several QTLs of varying effect sizes when coupling on the first three chromosomes and two QTLs with repulsion on the fourth chromosome (Table 1).

The choice of specific parameters in the simulated dataset, such as the number of individuals, chromosomes, and marker densities, aimed to emulate a realistic backcross scenario. The selection of 12 QTL with varying effect sizes reflects practical breeding scenarios, such as introgression programs, where traits are influenced by a limited number of loci with measurable effects. This design provides a controlled framework to systematically explore the impact of genetic architectures and marker densities on model performance under high-LD conditions. While a polygenic background was not included, this choice aligns with the study's focus on evaluating the models' abilities to handle marker correlation and variance partitioning in structured populations.

### 2.4 Empirical dataset

To complement our simulation studies and to validate the models using real-world data, we analyzed an existing mouse backcross dataset provided by Leiter et al. (2009). This dataset comprises a variable number of individuals (ranging from 142 to 310, depending on phenotypic data availability), 23 distinct phenotypes, and 311 SNP markers with genetic positions. The dataset is publicly accessible and can be found at https://phenome.jax.org/projects/Leiter2, last access: 20 May 2024. Notably, approximately 2.5 % of the marker genotypes were missing and were imputed using a hidden Markov model approach, implemented through the fill.geno function in R/qtl (Broman et al., 2003).

Variance components and heritabilities were estimated using the gremlin R package (Wolak, 2020) via restricted maximum likelihood. Standard errors for these estimates were obtained using the deltaSE function in gremlin, which applies the delta method to approximate variances. A separate application of the delta method was used to derive standard errors for Mendelian sampling variance, as detailed in Sect.  2.5.1.

### 2.5 Criteria for comparison

#### 2.5.1 Genetic and residual variances

To evaluate the models, we first calculated the variance in TBV (within-family) $\sigma_{g}^{2}$ for all 100 000 individuals. This was done by multiplying the simulated marker effects m with the covariance matrix, as per the method described by Bonk et al. (2016):

10 $$\sigma_{g}^{2}=m^{\prime }Rm.$$

In contrast, the variance in GEBVs ( $\sigma_{\hat{g}}^{2}$ ), which reflects how well the estimated marker effects mirror the true within-backcross genetic variability, was calculated for each replicate (500 individuals) of both G-BLUP and CAG-BLUP using estimated marker effects $\hat{m}$ instead. For GASC-BLUP and GASI-BLUP, $\sigma_{\hat{g}}^{2}={{\hat{m}}^{\prime }}_{\mathrm{sg}}R{\hat{m}}_{\mathrm{sg}}+{{\hat{m}}^{\prime }}_{\mathrm{nsg}}R{\hat{m}}_{\mathrm{nsg}}$ . The true residual variance ( $\sigma_{e}^{2}$ ) was obtained from the variance in residual effects across all individuals.

To evaluate model fit, we calculated the root-mean-square error (RMSE) of $\sigma_{\hat{g}}^{2}$ and the RMSE of the estimated residual variance ( ${\hat{\sigma }}_{e}^{2}$ ) across all 200 replications of each simulated scenario using

11 $$\begin{array}{l} {\mathrm{rMSE}}_{\sigma_{\hat{g}}^{2}}=\sqrt{\frac{1}{200}\sum_{i=1}^{500}{\left(\sigma_{\hat{g}}^{2^{\left(i\right)}}-\sigma_{g}^{2}\right)}^{2}}\\ {\mathrm{rMSE}}_{{\hat{\sigma }}_{e}^{2}}=\sqrt{\frac{1}{200}\sum_{i=1}^{500}{\left({\hat{\sigma }}_{e}^{2^{\left(i\right)}}-\sigma_{e}^{2}\right)}^{2}} \end{array},$$

where $\sigma_{\hat{g}}^{2^{\left(i\right)}}$ and ${\hat{\sigma }}_{e}^{2^{\left(i\right)}}$ denote the estimated genetic and residual variances of replicate i . Lower RMSE values indicate a closer approximation to the true variances and, hence, a better model fit.

Standard errors for $\sigma_{\hat{g}}^{2}$ were derived using a first-order Taylor expansion of its variance approximation considering the uncertainty in $\hat{m}$ . Specifically, the $\sigma_{\hat{g}}^{2}$ was computed as a function of $\hat{m}$ and R . The variance of $\hat{m}$ was obtained from the mixed model equations, leveraging the inverse coefficient matrix structure as described by Searle et al. (2006) and Lu and Shiou (2002). Standard errors were then computed as the square root of the derived variances.

#### 2.5.2 Accuracy of GEBVs and predictive ability

The accuracy was obtained as the correlation $\left(r_{\hat{g},g}\right)$ between the GEBVs and TBVs. A further criterion for the accuracy of the GEBVs was the RMSE of the GEBVs:

12 $${\mathrm{rMSE}}_{g}=\sqrt{\frac{1}{200}\frac{1}{500}\sum_{i=1}^{200}\sum_{j=1}^{500}{\left({\hat{g}}_{j}^{\left(i\right)}-g_{j}^{\left(i\right)}\right)}^{2}},$$

where ${\hat{g}}_{j}^{\left(i\right)}$ and $g_{j}^{\left(i\right)}$ denote the estimated GEBVs and TBVs of individual j in replicate i .

In GS, breeders often select individuals based on their GEBVs. As such, improved accuracy in GEBV estimation directly enhances selection accuracy. To evaluate the models' predictive capabilities, each replicate was treated as a training set, and its estimated marker effects were used to predict GEBVs for the remaining 199 replicates. This generated 99 500 GEBVs per replicate. The GEBVs were then paired with their corresponding TBV, and selection was based on GEBV at varying rates (from 5 % to 100 %). We then calculated the mean TBV of the selected animals.

#### 2.5.3 Cross-validation (4-fold)

We utilized 4-fold cross-validation to assess the models' prediction accuracies within our empirical dataset, a method chosen due to the limited number of individuals available (Kuhn and Johnson, 2013). In this approach, the dataset was evenly divided into four distinct subsets. During each cross-validation cycle, three subsets were combined to form the training set, while the remaining subset was designated as the validation set. The training sets were used to estimate predictor effects, which, in turn, predicted the GEBVs for individuals in the validation set. The accuracy of these predictions was evaluated based on the correlation between the predicted GEBVs and the observed phenotypes within the validation set. This cross-validation cycle was performed four times, rotating the validation set each time to ensure that each subset was used as the validation set once. The prediction accuracies obtained from each cycle were then averaged to produce a final estimate of model performance.

All analyses were conducted using the R package gremlin (Wolak, 2020) and custom scripts written in R version 4.2.2 (R Core Team, 2022).

## 3 Results

This section evaluates the performance of various genomic prediction models – CAG-BLUP, GASI-BLUP, GASC-BLUP, and the conventional G-BLUP – within a backcross population using both simulated and empirical datasets. The analyses focus on model performance across different trait genetic architectures, heritabilities, and marker densities.

### 3.1 Simulated dataset analysis

Our simulated dataset consisted of 18 scenarios, each defined by combinations of two trait genetic architectures (independent and dependent QTL), three heritability levels (0.17, 0.29, and 0.70), and three marker densities (2020, 420, and 220 markers). The evaluation of the models centered on additive genetic variability estimates, accuracy of GEBVs, and predictive ability.

#### 3.1.1 Model performance in terms of additive genetic variability estimates and accuracy of GEBVs

For independent QTL scenarios (Table 2 and Table S1 in the Supplement), G-BLUP and GASI-BLUP, under the assumption of marker independence, provided estimates for additive ( ${\hat{\sigma }}_{a}^{2}$ ) and residual ( ${\hat{\sigma }}_{e}^{2}$ ) variance components that were closer to the true variances than those provided by CAG-BLUP and GASC-BLUP. Across all scenarios, each model consistently underestimated $\sigma_{g}^{2}$ , a tendency that diminished as heritability increased (Table 2). This underestimation was significantly less severe in G-BLUP and GASI-BLUP compared to in CAG-BLUP and GASC-BLUP, respectively. Specifically, G-BLUP's underestimation of true variance ranged between 16 %–44 %, a marked improvement over CAG-BLUP, which exhibited a 20 %–47 % underestimation, while also enhancing GEBV prediction accuracy by 2 percentage points. Even more impressively, GASI-BLUP reduced the underestimation of true variance to 12 %–34 % and further improved GEBV prediction accuracy by approximately 2 percentage points relative to G-BLUP. This highlights GASI-BLUP's superior performance in both estimating variance and predicting GEBVs.

In scenarios involving dependent QTLs (Table 2), CAG-BLUP demonstrated an improved performance over G-BLUP in predicting $\sigma_{g}^{2}$ at lower heritabilities, indicating its utility in scenarios with dependent QTLs. The degree of underestimation for CAG-BLUP was observed to be between 24 %–32 %, a marked improvement when compared to G-BLUP, which exhibited a range of 25 %–35 % underestimation. Moreover, CAG-BLUP enhanced GEBV prediction accuracy by up to 2 percentage points compared to G-BLUP. However, as heritability increased to 70 %, the advantage of CAG-BLUP waned, with G-BLUP exhibiting a lower underestimation rate of 11 % compared to CAG-BLUP's 13 %, reversing the trend observed at lower heritability levels.

**Table 2:** Mean performance of the models in various scenarios of heritability in simulations of backcross populations.

Simulated			Model	Estimated parameters				
$h^{2}$	$\sigma_{g}^{2}$	$\;\sigma_{e}^{2}$		${\hat{\sigma }}_{a}^{2}$ (RMSE)	$\sigma_{\hat{g}}^{2}$ (RMSE)	${\hat{\sigma }}_{e}^{2}$ (RMSE)	Accuracy	RMSE of
							( $r_{\hat{g},g}$ )	GEBVs
Independent simulation								
0.17	3.27	16.00	G-BLUP	3.3(0.91)	1.83(1.58)	16.02(1.1)	0.77	1.18
			CAG-BLUP	4.31(1.83)	1.72(1.68)	16.8(1.34)	0.75	1.22
			GASI-BLUP	3.22(1.06)	2.15(1.31)	16.06(1.11)	0.79	1.13
			GASC-BLUP	6.77(7.07)	2.03(1.41)	16.66(1.28)	0.77	1.17
0.29	3.27	8.01	G-BLUP	3.25(0.63)	2.17(1.2)	8.04(0.57)	0.84	1
			CAG-BLUP	5.12(2.37)	2.06(1.31)	8.68(0.88)	0.82	1.06
			GASI-BLUP	3.15(0.75)	2.41(0.99)	8.08(0.58)	0.86	0.94
			GASC-BLUP	8.68(8.92)	2.3(1.09)	8.55(0.79)	0.84	1
0.70	3.27	1.40	G-BLUP	3.18(0.34)	2.74(0.59)	1.39(0.12)	0.94	0.62
			CAG-BLUP	9.67(6.7)	2.62(0.7)	1.72(0.35)	0.92	0.7
			GASI-BLUP	3.17(0.43)	2.87(0.49)	1.39(0.12)	0.95	0.56
			GASC-BLUP	26.16(25.35)	2.78(0.56)	1.44(0.21)	0.94	0.61
Dependent simulation								
0.17	6.54	31.93	G-BLUP	6.62(1.32)	4.27(2.5)	31.19(2.24)	0.82	1.48
			CAG-BLUP	8.99(3.08)	4.42(2.36)	32.35(2.13)	0.84	1.41
			GASI-BLUP	5.2(2.19)	5.72(1.46)	31.57(2.1)	0.93	0.99
			GASC-BLUP	13.05(15.01)	5.62(1.48)	32.18(2.09)	0.92	1.02
0.29	6.54	16.00	G-BLUP	6(1.03)	4.88(1.84)	15.54(1.18)	0.88	1.24
			CAG-BLUP	8.79(2.64)	4.96(1.76)	16.47(1.17)	0.89	1.2
			GASI-BLUP	4.57(2.32)	5.92(1.07)	15.87(1.06)	0.95	0.81
			GASC-BLUP	18.89(20.65)	5.85(1.1)	16.19(1.1)	0.95	0.84
0.70	6.54	2.80	G-BLUP	4.74(1.85)	5.81(0.84)	2.61(0.29)	0.96	0.76
			CAG-BLUP	9.61(3.4)	5.71(0.93)	3.15(0.42)	0.95	0.81
			GASI-BLUP	3.56(3.04)	6.21(0.57)	2.75(0.2)	0.98	0.54
			GASC-BLUP	39.51(35.98)	6.19(0.57)	2.58(0.34)	0.98	0.57

Note that $\sigma_{g}^{2}$ denotes true genetic (Mendelian sampling) variance; $\sigma_{e}^{2}$ denotes true residual variance; ${\hat{\sigma }}_{a}^{2}$ and ${\hat{\sigma }}_{e}^{2}$ denote mean estimated additive and residual variance components, respectively; $\sigma_{\hat{g}}^{2}$ denotes mean estimated genetic (Mendelian sampling) variance; RMSE denotes root mean squared error, indicating the deviation of estimated variances from true values; $h^{2}$ denotes heritability; GEBVs denotes genomic estimated breeding values; and accuracy ( $r_{\hat{g},g}$ ) refers to the correlation between GEBVs and true breeding values, indicating the precision of GEBVs in predicting true breeding values. Results are for a marker density of 2020.

GASI-BLUP exhibited a consistently strong performance across varying levels of heritability, effectively reducing the underestimation of the true variance more efficiently than its counterparts. In particular, GASI-BLUP decreased the underestimation to 5 %–12 %, a reduction notably greater than that observed with G-BLUP, which ranged from 11 % to 35 %. Additionally, GASI-BLUP improved the accuracy of GEBVs by as much as 11 percentage points relative to G-BLUP. Although GASC-BLUP consistently outperformed both CAG-BLUP and G-BLUP in reducing the degree of underestimation, its performance did not exceed that of GASI-BLUP despite being specifically designed for scenarios involving dependent QTLs. The degree of underestimation attributable to GASC-BLUP was recorded to be between 5 %–14 %, signifying an enhancement relative to G-BLUP and CAG-BLUP but still falling short of the superior efficiency demonstrated by GASI-BLUP.

The performance of the models under varying marker densities (Table 3) paralleled the trends observed under varying heritabilities for both independent and dependent QTL scenarios (Table 2). With an increase in marker densities, improvements were noted in $\sigma_{g}^{2}$ , the accuracy of GEBVs, and the RMSE of GEBV predictions. However, these improvements were more modest in comparison to those driven by heritability changes. Specifically, in the context of G-BLUP's performance in independent QTL scenarios, an increase in marker densities resulted in a 1 percentage point reduction in the underestimation of true genetic variance and a 1 % increase in GEBV accuracy. In contrast, an increase in heritability led to a significant 27 % decrease in the underestimation of true genetic variance and a substantial increase of 17 percentage points in GEBV accuracy. See Table S1 in the Supplement for a detailed exploration of the performance of these genomic prediction models across other scenarios of heritability and marker density.

**Table 3:** Mean performance of the models in various scenarios of marker density in simulations of backcross populations.

Simulated			Model	Estimated parameters				
MD	$\sigma_{g}^{2}$	$\;\sigma_{e}^{2}$		${\hat{\sigma }}_{a}^{2}$ (RMSE)	$\sigma_{\hat{g}}^{2}$ (RMSE)	${\hat{\sigma }}_{e}^{2}$ (RMSE)	Accuracy	RMSE of
							( $r_{\hat{g},g}$ )	GEBVs
Independent simulation								
220	3.27	8.01	G-BLUP	3.08(0.63)	2.12(1.25)	8.14(0.6)	0.83	1.02
			CAG-BLUP	4.93(2.14)	2.04(1.33)	8.67(0.88)	0.81	1.06
			GASI-BLUP	2.89(0.76)	2.36(1.03)	8.19(0.61)	0.85	0.96
			GASC-BLUP	8.21(7.63)	2.28(1.11)	8.55(0.79)	0.83	1.01
420	3.27	8.01	G-BLUP	3.2(0.63)	2.15(1.22)	8.06(0.58)	0.84	1
			CAG-BLUP	5.07(2.3)	2.06(1.31)	8.67(0.87)	0.82	1.06
			GASI-BLUP	3.04(0.73)	2.39(1.01)	8.11(0.58)	0.86	0.95
			GASC-BLUP	8.41(7.76)	2.29(1.1)	8.55(0.79)	0.84	1
2020	3.27	8.01	G-BLUP	3.25(0.63)	2.17(1.2)	8.04(0.57)	0.84	1
			CAG-BLUP	5.12(2.37)	2.06(1.31)	8.68(0.88)	0.82	1.06
			GASI-BLUP	3.15(0.75)	2.41(0.99)	8.08(0.58)	0.86	0.94
			GASC-BLUP	8.68(8.92)	2.3(1.09)	8.55(0.79)	0.84	1
Dependent simulation								
220	6.54	16.00	G-BLUP	5.79(1.12)	4.8(1.91)	15.68(1.15)	0.87	1.27
			CAG-BLUP	8.64(2.48)	4.91(1.79)	16.46(1.18)	0.88	1.22
			GASI-BLUP	4.12(2.62)	5.87(1.09)	16.04(1.07)	0.94	0.85
			GASC-BLUP	16.79(17.16)	5.84(1.11)	16.2(1.1)	0.94	0.87
420	6.54	16.00	G-BLUP	5.93(1.07)	4.84(1.87)	15.58(1.17)	0.88	1.25
			CAG-BLUP	8.73(2.57)	4.94(1.77)	16.47(1.18)	0.88	1.21
			GASI-BLUP	4.34(2.45)	5.9(1.08)	15.93(1.06)	0.95	0.82
			GASC-BLUP	18.59(20.22)	5.85(1.11)	16.18(1.1)	0.95	0.85
2020	6.54	16.00	G-BLUP	6(1.03)	4.88(1.84)	15.54(1.18)	0.88	1.24
			CAG-BLUP	8.79(2.64)	4.96(1.76)	16.47(1.17)	0.89	1.2
			GASI-BLUP	4.57(2.32)	5.92(1.07)	15.87(1.06)	0.95	0.81
			GASC-BLUP	18.89(20.65)	5.85(1.1)	16.19(1.1)	0.95	0.84

Note that $\sigma_{g}^{2}$ denotes true genetic (Mendelian sampling) variance; $\sigma_{e}^{2}$ denotes true residual variance; ${\hat{\sigma }}_{a}^{2}$ and ${\hat{\sigma }}_{e}^{2}$ denote mean estimated additive and residual variance components, respectively; $\sigma_{\hat{g}}^{2}$ denotes mean estimated genetic (Mendelian sampling) variance; RMSE denotes root mean squared error, indicating the deviation of estimated variances from true values; MD denotes marker density; GEBVs denotes genomic estimated breeding values; and accuracy ( $r_{\hat{g},g}$ ) refers to the correlation between GEBVs and true breeding values, indicating the precision of GEBVs in predicting true breeding values. Results are for a heritability of 0.29.

#### 3.1.2 Estimates of marker effects

**Figure 1:**
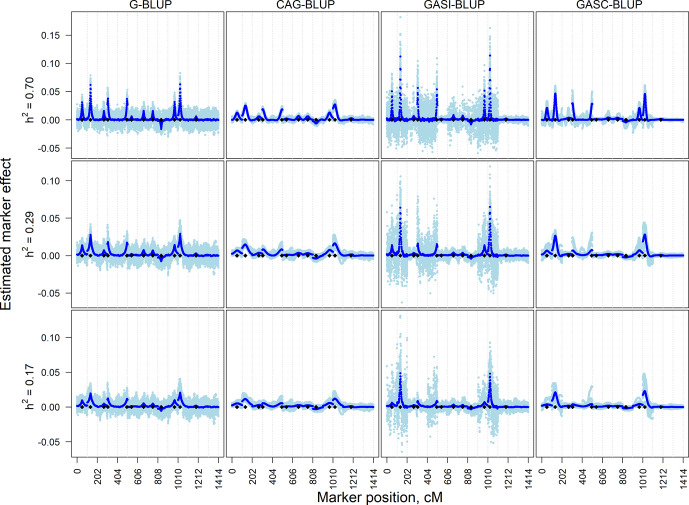
Estimated marker effects of 20 randomly selected replicates (light blue) and the means of all 200 replicates (black) in independent simulation scenarios with a marker density of 2020 markers and different heritabilities ( $h^{2}$ ). The black diamonds are the quantitative trait locus positions. The results presented are for chromosomes 1–14 only.

**Figure 2:**
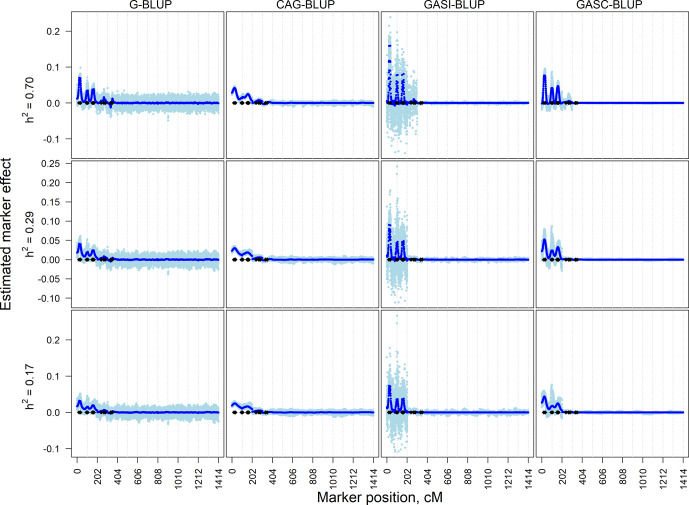
Estimated marker effects of 20 randomly selected replicates (light blue) and the means of all 200 replicates (black) in the dependent simulation scenarios with a marker density of 2020 markers and different heritabilities ( $h^{2}$ ). The black diamonds are the quantitative trait locus positions. The results presented are for chromosomes 1–6 only.

**Figure 3:**
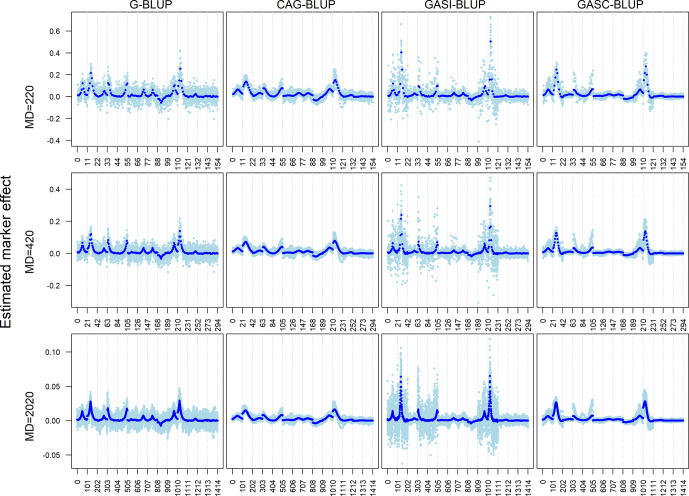
Estimated marker effects of 20 randomly selected replicates (light blue) and the means of all 200 replicates (black) in independent simulation scenarios with a heritability of 0.29 and different marker densities (MDs). The results presented are for chromosomes 1–14 only.

Our analysis, presented in Figs. 1–3, evaluated the performance of the models in estimating marker effects across independent and dependent QTL simulations, using data from 20 randomly selected replicates and all 200 replicates.

In the independent simulation, the magnitude of estimated marker effects increased with heritability, while variation was more pronounced at lower heritability levels (Fig. 1). G-BLUP and GASI-BLUP showed higher noise, particularly on chromosomes without QTLs or with small QTL effects. Conversely, CAG-BLUP and GASC-BLUP, benefiting from a correlation structure, produced smoother curves across chromosomes, aiding in the identification of QTL regions. GASI-BLUP and GASC-BLUP exhibited larger marker effects on chromosomes with QTLs due to unequal variance allocation among markers.

The dependent simulation revealed similar trends (Fig. 2). G-BLUP and GASI-BLUP displayed pronounced marker effects at QTL positions, while CAG-BLUP and GASC-BLUP generated smoother curves over dependent QTL. The correlation structure in CAG-BLUP and GASC-BLUP enhanced QTL detection but limited their ability to identify closely positioned dependent QTLs. These trends were consistent across scenarios with different marker densities.

The models' performances under varying marker densities resembled their performances under different heritabilities. However, a notable difference emerged in terms of the magnitude of marker effects, which decreased as marker densities increased (Fig. 3). This contrasted with the effect of heritability, where marker effects increased with higher heritability levels.

#### 3.1.3 Predictive ability

**Figure 4:**
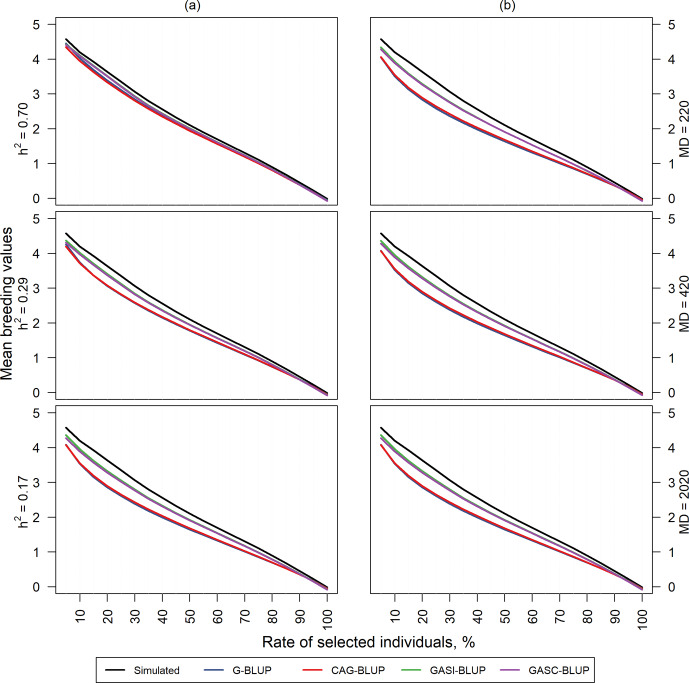
Mean TBVs of individuals that were selected by their estimated breeding values in the dependent simulation. Panel **(a)** shows results for scenarios with a marker density of 2020 markers and different heritabilities ( $h^{2}$ ). Panel **(b)** shows results for scenarios with a heritability of 0.17 and different marker densities (MDs).

In the evaluation of predictive ability, we focused on the mean TBVs of individuals selected based on their GEBVs within the dependent simulation. Figure 4a visually presents this assessment, comparing the mean TBVs of selected individuals for each model to a reference line representing individuals selected based on their TBV.

Notably, as heritability increased, the mean TBVs of selected individuals tended to approach the reference line for all models. Specifically, GASI-BLUP and GASC-BLUP demonstrated not only comparable but also superior performance compared to G-BLUP and CAG-BLUP. Interestingly, G-BLUP and CAG-BLUP exhibit similar levels of performance in relation to each other.

In contrast, the influence of marker distance on the rate of individuals selected based on their GEBVs is less pronounced, as shown in Fig. 4b. Heritability significantly impacts the predictive ability of the models, while the effect of marker distance on the selection rate is relatively subtle. This underscores the primary role of heritability in determining predictive accuracy, with marker distance playing a secondary role in this context.

### 3.2 Empirical data analysis

The analysis of empirical data from a mouse backcross study, alongside the simulated dataset, aimed to assess the real-world applicability of various genomic prediction models. This evaluation compared their performance, focusing on traits like estimated genetic variance, heritability, and prediction accuracy, as detailed in Table 4. GASI-BLUP and GASC-BLUP consistently demonstrated superior performance over G-BLUP and CAG-BLUP in terms of these metrics. Notably, GASC-BLUP provided the highest estimates of additive genetic variance for each trait and matched GASI-BLUP in reaching the highest levels of prediction accuracy.

**Table 4:** Performance of the models in real backcross data.

Model	${\hat{\sigma }}_{a}^{2}$ (SE)	$\sigma_{\hat{g}}^{2}$ (SE)	${\hat{\sigma }}_{e}^{2}$ (SE)	${\hat{h}}^{2}$ (SE)	Accuracy
Bone mineral content					
G-BLUP	8.37 × 10^−4^ (3.99 × 10^−4^)	4.95 × 10^−4^ (1.59 × 10^−4^)	2.64 × 10^−3^ (3.8 × 10^−4^)	0.16(0.05)	0.38
CAG-BLUP	1.06 × 10^−3^ (5.62 × 10^−4^)	5.86 × 10^−4^ (1.95 × 10^−4^)	2.77 × 10^−3^ (3.58 × 10^−4^)	0.17(0.05)	0.39
GASI-BLUP	6.46 × 10^−4^ (3.94 × 10^−4^)	5.41 × 10^−4^ (2.01 × 10^−4^)	2.73 × 10^−3^ (3.85 × 10^−4^)	0.17(0.05)	0.41
GASC-BLUP	8.65 × 10^−4^ (6.56 × 10^−4^)	6.20 × 10^−4^ (2.21 × 10^−4^)	2.82 × 10^−3^ (3.61 × 10^−4^)	0.18(0.05)	0.42
Total cholesterol in plasma					
G-BLUP	190.22(58.29)	144.35(26.13)	154.32(25.78)	0.48(0.06)	0.61
CAG-BLUP	382.01(149.22)	152.63(29.69)	181.50(26.01)	0.46(0.06)	0.61
GASI-BLUP	187.30(82.26)	170.74 (30.75)	162.10(24.86)	0.51(0.06)	0.67
GASC-BLUP	1064.19(743.44)	186.18(32.36)	169.57(25.83)	0.52(0.05)	0.67
High-density lipoproteins					
G-BLUP	160.01(49.75)	119.25(22.49)	135.29(22.51)	0.47(0.06)	0.58
CAG-BLUP	335.51(131.18)	130.01(26.02)	156.54(22.50)	0.45(0.06)	0.59
GASI-BLUP	171.89(78.74)	140.84(25.90)	140.12(21.64)	0.50(0.06)	0.65
GASC-BLUP	877.61(626.22)	151.67(27.46)	149.62(22.58)	0.50(0.06)	0.65

Note that ${\hat{\sigma }}_{a}^{2}$ and ${\hat{\sigma }}_{e}^{2}$ denote estimated additive and residual variance; $\sigma_{\hat{g}}^{2}$ denotes estimated genetic (Mendelian sampling) variance; ${\hat{h}}^{2}$ denotes heritability ( $\sigma_{\hat{g}}^{2}/\left(\sigma_{\hat{g}}^{2}+{\hat{\sigma }}_{e}^{2}\right)$ ); and SE denotes standard error, presented in parentheses.

**Figure 5:**
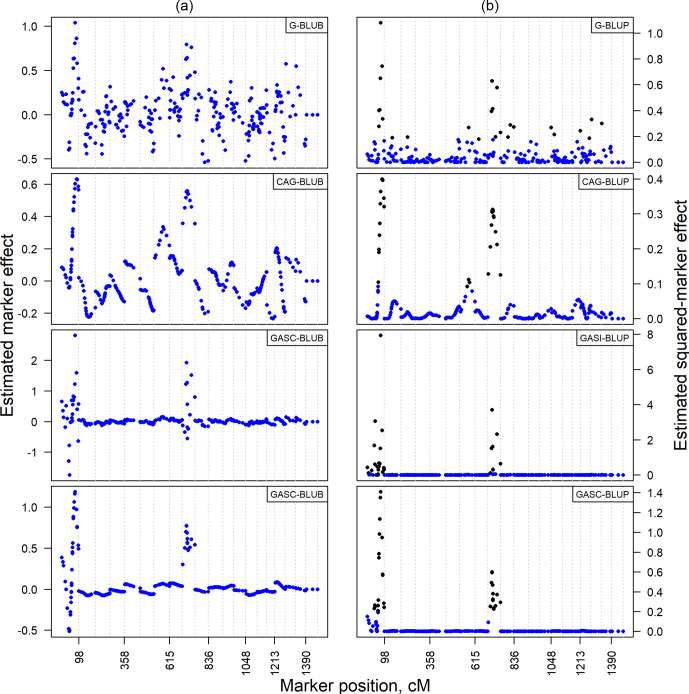
Estimated marker effect **(a)** and squared-marker effect **(b)** for cholesterol in empirical backcross data. Black dots indicate the top 10 % of markers with the largest effects.

The analysis of estimated marker effects for cholesterol levels, illustrated in Fig. 5a, confirmed patterns observed in the simulated data. Absolute values, with the top 10 % of markers highlighted in black, displayed fewer fluctuations in Fig. 5b compared to non-absolute values in Fig. 5a. Notably, the G-BLUP model, which had markers in the top 10 % spread across 10 chromosomes, was identified as the model with the highest variability. Conversely, other models showed a more focused distribution of their top 10 % of markers, limited to no more than three chromosomes. This highlighted clear differences in model performance and marker detection capability across chromosomes in an empirical setting, reflecting similar trends noted in dependent QTL scenarios of the simulated data.

## 4 Discussion

### 4.1 Model development and assumptions

In our study, we have developed three novel genomic prediction models, namely CAG-BLUP, GASI-BLUP, and GASC-BLUP, each tailored to overcome specific limitations identified in traditional models like G-BLUP. Traditional GS models often do not fully capture the complexities of genetic architecture, particularly in backcross populations, due to their assumptions of marker independence and uniform variance contribution across markers. Our models offer a nuanced approach to address these complexities.

CAG-BLUP is designed with the specific intent of enhancing prediction accuracy for traits influenced by dependent QTLs. It utilizes a covariance matrix to account for marker correlations resulting from LD, drawing upon the methodology developed by Bonk et al. (2016) for full sibs. This model aims to improve prediction accuracy in genetic architectures where LD and marker correlations are pivotal in accurate genetic variance estimation.

GAS-BLUP models, comprising GASI-BLUP for scenarios with independent markers and GASC-BLUP for those with correlated markers, mark a departure from traditional assumptions by recognizing the unequal contributions of markers to genetic variance. The introduction of two distinct shrinkage parameters in these models is a strategic innovation aimed at balancing the need for computational efficiency with the methodological demand for precision. This dual-parameter approach is informed by the theoretical ideal of assigning unique shrinkage values to each chromosome. Due to computational limitations, however, the models pragmatically apply one parameter to chromosomes marked by significant QTL presence and another to the remainder. This differentiation allows for a more detailed analysis of genomic regions, enhancing the model's capacity to reflect trait heritability.

By integrating considerations for marker correlation, along with the nuanced application of shrinkage parameters, our models are specifically engineered to enhance the accuracy of genomic predictions within backcross populations. This development signifies a targeted effort to refine GS practices, offering a set of tools designed to accurately model the genetic dynamics encountered in such breeding schemes.

### 4.2 Model performance analysis

The comparative analysis of the newly developed models (CAG-BLUP, GASI-BLUP, and GASC-BLUP) against the traditional G-BLUP model highlighted significant variances in their ability to estimate additive genetic variance and the accuracy of GEBVs. This section synthesizes these findings, emphasizing the implications of distinct model assumptions for genomic prediction accuracy, particularly within backcross populations.

#### 4.2.1 Additive genetic variability and GEBV accuracy

Our comparative analysis revealed significant discrepancies in the ${\hat{\sigma }}_{a}^{2}$ values across the models, underscoring the influence of their distinct assumptions on genomic predictions. It is crucial to note that this variance is of limited relevance within backcross populations as it represents genetic variability among unrelated, non-inbred individuals in the base population (Legarra, 2016).

More pertinent to backcross populations is the $\sigma_{g}^{2}$ or the Mendelian sampling variance, which directly reflects the models' abilities to accurately capture within-family genetic variability. Our analysis demonstrated significant disparities in $\sigma_{g}^{2}$ estimates among the models, with G-BLUP and GASI-BLUP showing greater proficiency in scenarios characterized by independent QTLs compared to CAG-BLUP and GASC-BLUP, respectively. These models, adhering to the assumption of marker independence, yielded estimates more closely aligned with actual genetic variance, evidencing their efficacy in representing additive genetic effects with accuracy.

Conversely, CAG-BLUP's design, incorporating LD and marker correlations, proved to be advantageous in scenarios with dependent QTLs and lower heritabilities. However, as heritability increases, the benefit conferred by CAG-BLUP is diminished, suggesting a convergence towards direct additive genetic influences in environments marked by high heritability.

Notably, our analysis indicated the superior performance of GAS-BLUP models compared to G-BLUP and CAG-BLUP models. However, despite its intention to address marker correlations, GASC-BLUP did not surpass GASI-BLUP, even in simulations involving dependent QTL architectures. This suggests a potential limitation in GASC-BLUP's current strategy for handling marker correlations, implying that it may not fully capture the complexities inherent in such genetic architectures. Furthermore, sensitivities in detecting chromosome-carrying QTLs (see Table S2) could also contribute to this observation. Specifically, an examination of QTL detection capabilities, particularly between G-BLUP and CAG-BLUP, revealed a slight advantage for G-BLUP in identifying chromosomes carrying QTLs. This disparity in QTL detection might contribute to the observed performance differences, underscoring the necessity for further refinement in GASC-BLUP's approach to enhance its effectiveness in elucidating the dynamics of dependent QTLs.

#### 4.2.2 Influence of heritability and marker density

This study further explored the impact of heritability and marker density on the accuracy of GEBVs and the estimation of genetic variance. A consistent trend of underestimating true genetic variance was observed across all models, a pattern that lessened with increases in heritability and marker density. These findings are consistent with prior research (Dou et al., 2016; Peixoto et al., 2016; Poland et al., 2012), suggesting that a limited number of markers can effectively represent genetic variability in populations like backcross or F2 due to high allele sharing. These insights emphasize the necessity of strategically considering both heritability and marker density in genomic prediction efforts, highlighting their significant influence on the outcomes of genomic predictions.

#### 4.2.3 Estimates of marker effects

Our examination of the genomic prediction models' strategies for estimating marker effects unveils significant distinctions in their methodologies, each bearing consequential implications for the accuracy of GS and the delineation of QTLs.

*Marker independence vs. correlation structures*. The analysis presented in Figs. 1–3 demonstrated the divergent approaches taken by the models under review. G-BLUP and GASI-BLUP, operating under the assumption of marker independence, exhibited a propensity to generate estimates with notable variability. This variability was particularly pronounced on chromosomes devoid of clear QTLs or those with minimal QTL effects, potentially making their identification difficult. Such variability might impede the clear distinction between true genetic signals and background noise, aligning with observations by Kärkkäinen and Sillanpää (2012) regarding the implications of noise in genetic marker data for QTL detection.

Conversely, models like CAG-BLUP and GASC-BLUP that integrate correlation structures into their estimation processes produce markedly smoother estimates across the genome, facilitating the identification of QTL regions, particularly in genetic contexts characterized by QTL dependency. However, these models may not pinpoint closely situated dependent QTLs accurately, tending to generate broader curves over areas rich in QTLs rather than identifying specific loci precisely. This limitation indicates the need for further refinement to fully resolve the complexities of tightly linked QTL clusters. The observed effects in a randomly chosen replicate from both independent and dependent simulations (Fig. S1a and b) depict the impacts of integrating correlation structures and of unequal variance allocation on QTL identification. While these approaches significantly refine the estimation of marker effects, delineating the precise locations of QTLs ultimately requires the application of fine mapping techniques.

*Shrinkage and its implications*. The variation observed in the magnitude of estimated marker effects across the models can largely be ascribed to the models' differential applications of shrinkage to random effects. Shrinkage, a process influenced by trait heritability, genetic architecture, and marker density, is pivotal in striking a balance between the accuracy and precision of marker effect estimation. Supported by several works (Habier et al., 2011; De Los Campos et al., 2009; Meuwissen et al., 2001; Xu, 2003), our findings reveal a more pronounced shrinkage towards zero in CAG-BLUP's estimated marker effects compared to in those of G-BLUP. This difference is likely to reflect variations in the underlying shrinkage parameters, which are determined by the variance components (see ${\hat{\sigma }}_{a}^{2}$ and ${\hat{\sigma }}_{e}^{2}$ in Tables 2 and 3).

### 4.3 Integration of covariance structures

The use of covariance or correlation structures to account for marker associations in GS is being increasingly recognized for its potential to enhance genetic prediction accuracy (Gianola et al., 2003; Martínez et al., 2017; Ramstein et al., 2016; Wittenburg et al., 2016; Yang and Tempelman, 2012). This approach, by acknowledging the complex interactions among genetic markers, moves beyond the limitations of traditional models that assume marker independence and uniform variance. Our study leverages a covariance matrix, developed by Bonk et al. (2016), that is grounded in genetic principles and that utilizes Haldane's mapping function (Haldane, 1919) to model LD between markers effectively. While this methodology is similar to that proposed by Mathew et al. (2017), it differs by avoiding iterative estimation methods, instead relying on a direct application of robust genetic insights to estimate LD decay.

### 4.4 Equivalent models

Our study introduces the CAG-BLUP, GASI-BLUP, and GASC-BLUP models, along with their marker-based equivalents (see Appendices), highlighting the computational flexibility that is crucial for addressing the diverse datasets in contemporary breeding programs. These models provide equivalent predictive accuracy, allowing for strategic application based on dataset characteristics – GRM-based models for datasets with a high marker-to-individual ratio and marker-based models for scenarios with more individuals than markers. This dual-strategy approach caters to the computational needs of geneticists and breeders, ensuring the models' applicability across various genomic prediction scenarios. The adaptability of our models, underscored by their computational efficiency and the robustness in capturing genetic architecture, enhances the practical utility of GS in breeding and research, facilitating faster and more effective genetic improvement efforts.

### 4.5 Empirical data validation

Validation against empirical data from a mouse backcross study underscores the practical applicability of our models, particularly in predicting genetic traits like cholesterol levels. The observed variability in model performance highlights the importance of model selection based on the genetic architecture and heritability of the trait of interest. These real-world applications confirm the models' robustness and underscore the potential for their integration into current breeding programs, paving the way for more precise genetic improvements.

### 4.6 Limitations and future directions

Despite the clear benefits shown by our proposed models, several factors may limit their immediate generalizability and highlight potential avenues for further research. First, we focused on a controlled backcross (BC1) populations derived from inbred parents, an approach that simplifies the underlying LD structure but that may not capture the allelic diversity of more advanced or heterogeneous populations. Second, by centering primarily on additive effects and a finite number of QTLs, we have not explored how dominance or epistasis might influence genomic predictions, and we have not assessed the performance of our models under fully polygenic architectures. Addressing these interactions could be especially important for complex traits governed by numerous small-effect loci. Third, while incorporating marker correlation and unequal shrinkage has proven to be advantageous in both simulated and empirical data, the ideal calibration of these parameters across multiple chromosomes remains computationally demanding and will require refinement for large-scale breeding programs.

Moving forward, extending these methods to later backcross generations, multi-parent designs, and introgression schemes will provide a more comprehensive evaluation of their utility. Incorporating non-additive genetic effects – such as dominance and epistasis – could offer a more realistic depiction of many agriculturally relevant traits. Further optimization of shrinkage parameterization, potentially adopting chromosome-specific or segment-specific approaches, could also enhance the models' accuracy. Empirical validations in species with different genome complexities and varying population structures will ultimately clarify how well these methodologies scale and adapt to diverse breeding scenarios. By systematically addressing these limitations, future research can strengthen the practical utility of CAG-BLUP, GASI-BLUP, and GASC-BLUP, ensuring that their potential benefits translate into improved genetic gains across a broader spectrum of breeding programs.

## 5 Conclusions

In this study, we introduced three new genomic prediction models – CAG-BLUP, GASI-BLUP, and GASC-BLUP – specifically tailored for backcross populations and demonstrated their superiority over the traditional G-BLUP model by more accurately accounting for genetic architecture, heritability, and marker density. GASI-BLUP excelled in scenarios with independent QTLs, leveraging unequal variance allocation, while CAG-BLUP proved to be effective for dependent QTLs and lower heritabilities through marker correlation consideration. Validated with both simulated and empirical data, these models affirm the critical role of model selection in enhancing genomic prediction accuracy, offering significant advancements for breeding programs. This research underscores the necessity of developing tailored GS methodologies to improve genetic gains and to ensure food security, laying the groundwork for future explorations to broaden model applications and to integrate comprehensive genetic effects.

## Supplement

10.5194/aab-68-377-2025-supplement The supplement related to this article is available online at https://doi.org/10.5194/aab-68-377-2025-supplement.

## Data Availability

The data used and analyzed during this study are available from the corresponding author upon request.

## Appendix A Equivalent SNP-BLUP model

This Appendix presents the equivalent SNP-BLUP model of the mixed linear model described in Eq. (3) in the main text. The equivalence is crucial for understanding the computational efficiency and theoretical underpinnings of the CAG-BLUP model. The equivalent SNP-BLUP model for the mixed linear model (Eq. 3) is expressed as follows:

A1
y=1μ+Zm+e,

where y , 1 , μ , and e are as previously defined in Eq. (3); m is a p×1 vector of marker effects; and Z is an n×p design matrix allocating records to marker effects. Further, $m∼N\left(0,R\sigma_{m}^{2}\right)$ , where $\sigma_{m}^{2}$ is the variance component of marker effects, and R is the corresponding covariance matrix. For an independent marker approach, we consider $m∼N\left(0,I\sigma_{m}^{2}\right)$ .

The estimates for μ and m can be computed by solving

A2
$$\left[\begin{array}{c} \mu \\ \hat{m} \end{array}\right]={\left[\begin{array}{cc} 1^{\prime }1 & 1^{\prime }Z\\ Z^{\prime }1 & Z^{\prime }Z+R^{-1}\cdot \sigma_{e}^{2}/\sigma_{m}^{2} \end{array}\right]}^{-1}\left[\begin{array}{c} 1^{\prime }y\\ Z^{\prime }y \end{array}\right].$$

The GEBVs can be obtained with

A3
$$\hat{g}=Z\hat{m}.$$

## Appendix B Proof of equivalence of linear models

This Appendix provides a detailed proof demonstrating the equivalence of the mixed linear models described in Eq. (3) and of the SNP-BLUP model (Eq. A1) from Appendix A. The proof is critical for validating the assumptions and results presented in the main text.

According to Henderson (1985), two linear models are equivalent if the $E\left(y\right)$ and variance Var $\left(y\right)$ of observations are identical. Consider the following:

B1y=1μ+Wg+e,B2y=1μ+Zm+e.

Both models have the same expected value, $E\left(y\right)=1\mu$ .

To demonstrate the equality of variances, we use the following identity:

B3
$$\begin{array}{rl} \sigma_{a}^{2} & =G_{\mathrm{CAG}}^{-1}\cdot G_{\mathrm{CAG}}\cdot \sigma_{a}^{2}\\ & =G_{\mathrm{CAG}}^{-1}\cdot \mathrm{Var}\left(g\right)\\ & =G_{\mathrm{CAG}}^{-1}\cdot \mathrm{Var}\left(Z\cdot m\right)\\ & =G_{\mathrm{CAG}}^{-1}\cdot Z\cdot R\cdot Z^{\prime }\cdot \sigma_{m}^{2}\\ & =G_{\mathrm{CAG}}^{-1}\cdot \frac{Z\cdot R\cdot Z^{\prime }}{s}\cdot s\cdot \sigma_{m}^{2}\\ & =s\cdot \sigma_{m}^{2}. \end{array}$$

Given W=I , the design matrix is as follows:

B4
$$\begin{array}{rl} \mathrm{Var}(Wg) & ={\mathrm{WG}}_{\mathrm{CAG}}W^{\prime }\cdot \delta_{a}^{2}\\ & =G_{\mathrm{CAG}}\cdot \delta_{a}^{2}\\ & =({\mathrm{ZRZ}}^{\prime })/s\cdot \delta_{a}^{2}\\ & ={\mathrm{ZRZ}}^{\prime }\cdot \delta_{m}^{2}\\ & =\mathrm{Var}(Zm). \end{array}$$

## Appendix C Equivalent SNP-BLUP model for GASC-BLUP

This Appendix presents the equivalent SNP-BLUP model for the GASC-BLUP method. The equivalence here is critical for understanding the relationship between GASC-BLUP and its SNP-level counterpart, highlighting the model's flexibility and adaptability.

The equivalent SNP-BLUP model for GASC-BLUP is formulated as follows:

C1
$$y=1\mu +Z_{\mathrm{sg}}m_{\mathrm{sg}}+Z_{\mathrm{nsg}}m_{\mathrm{nsg}}+e,$$

where $m_{\mathrm{sg}}$ and $m_{\mathrm{nsg}}$ are vectors of marker effects for significant and non-significant genome segments, respectively. $Z_{\mathrm{sg}}$ and $Z_{\mathrm{nsg}}$ are design matrices allocating records to the marker effects of these segments. $m_{\mathrm{sg}}∼N\left(0,R_{\mathrm{sg}}\sigma_{m_{\mathrm{sg}}}^{2}\right)$ and $m_{\mathrm{nsg}}∼N\left(0,R_{\mathrm{nsg}}\sigma_{m_{\mathrm{nsg}}}^{2}\right)$ , where $R_{\mathrm{sg}}$ and $R_{\mathrm{nsg}}$ are the covariance matrices, and $\sigma_{m_{\mathrm{sg}}}^{2}$ and $\sigma_{m_{\mathrm{nsg}}}^{2}$ are the variance components of marker effects for significant and non-significant genome segments, respectively. For GASI-BLUP, we consider $m_{\mathrm{sg}}∼N\left(0,I\sigma_{m_{\mathrm{sg}}}^{2}\right)$ and $m_{\mathrm{nsg}}∼N\left(0,I\sigma_{m_{\mathrm{nsg}}}^{2}\right)$ .

The estimates for μ , $m_{\mathrm{sg}}$ , and $m_{\mathrm{nsg}}$ can be computed by solving the following:

$$\begin{array}{rl} & \left[\begin{array}{c} \mu \\ {\hat{m}}_{\mathrm{sg}}\\ {\hat{m}}_{\mathrm{nsg}} \end{array}\right]=\\ & {\left[\begin{array}{ccc} 1^{\prime }1 & 1^{\prime }Z_{\mathrm{sg}} & 1^{\prime }Z_{\mathrm{nsg}}\\ {Z^{\prime }}_{\mathrm{sg}}1 & {Z^{\prime }}_{\mathrm{sg}}Z_{\mathrm{sg}}+R_{\mathrm{sg}}\cdot \left(\sigma_{e}^{2}/\sigma_{m_{\mathrm{sg}}}^{2}\right) & {Z^{\prime }}_{\mathrm{sg}}Z_{\mathrm{nsg}}\\ {Z^{\prime }}_{\mathrm{nsg}}1 & {Z^{\prime }}_{\mathrm{nsg}}Z_{\mathrm{sg}} & {Z^{\prime }}_{\mathrm{nsg}}Z_{\mathrm{nsg}}+R_{\mathrm{nsg}}\cdot \left(\sigma_{e}^{2}/\sigma_{m_{\mathrm{nsg}}}^{2}\right) \end{array}\right]}^{-1}\\ & \left[\begin{array}{c} 1^{\prime }y\\ {Z^{\prime }}_{\mathrm{sg}}y\\ {Z^{\prime }}_{\mathrm{nsg}}y \end{array}\right]. \end{array}$$

GEBVs are calculated by summing marker effects:

C2
$$\begin{array}{rl} {\hat{g}}_{\mathrm{sg}} & =Z_{\mathrm{sg}}{\hat{m}}_{\mathrm{sg}}\\ {\hat{g}}_{\mathrm{nsg}} & =Z_{\mathrm{nsg}}{\hat{m}}_{\mathrm{nsg}}. \end{array}$$

## Appendix D Equivalence of GASC-BLUP and its SNP-BLUP model

This Appendix demonstrates the theoretical equivalence between GASC-BLUP and its corresponding SNP-BLUP model. Establishing this equivalence is essential for validating the consistency and applicability of GASC-BLUP in genomic prediction analysis, particularly in the context of complex genetic architectures. To establish the equivalence between GASC-BLUP and its SNP-BLUP model, consider the following mixed linear models:

D1y=1μ+Wgsg+Wgnsg+e,D2y=1μ+Zsgmsg+Znsgmnsg+e.

Both models share the same expected value, $E\left(y\right)=1\mu$ . For equivalence in variances, we use the following identity:

$$\begin{array}{rl} \sigma_{a_{\mathrm{sg}}}^{2} & =G_{{\mathrm{CAG}}_{\mathrm{sg}}}^{-1}\cdot G_{{\mathrm{CAG}}_{\mathrm{sg}}}\cdot \sigma_{a_{\mathrm{sg}}}^{2}\\ & =G_{{\mathrm{CAG}}_{\mathrm{sg}}}^{-1}\cdot \mathrm{Var}\left(g_{\mathrm{sg}}\right)\\ & =G_{{\mathrm{CAG}}_{\mathrm{sg}}}^{-1}\cdot \mathrm{Var}\left(Z_{\mathrm{sg}}\cdot m_{\mathrm{sg}}\right)\\ & =G_{{\mathrm{CAG}}_{\mathrm{sg}}}^{-1}\cdot Z_{\mathrm{sg}}\cdot R_{\mathrm{sg}}\cdot {Z^{\prime }}_{\mathrm{sg}}\cdot \sigma_{m_{\mathrm{sg}}}^{2}\\ & =G_{{\mathrm{CAG}}_{\mathrm{sg}}}^{-1}\cdot \frac{Z_{\mathrm{sg}}\cdot R_{g}\cdot {Z^{\prime }}_{\mathrm{sg}}}{s_{\mathrm{sg}}}\cdot s_{\mathrm{sg}}\cdot \sigma_{m_{\mathrm{sg}}}^{2}\\ & =s_{\mathrm{sg}}\cdot \sigma_{m_{\mathrm{sg}}}^{2}. \end{array}$$

Considering W=I , we derive the following variance:

$$\begin{array}{rl} \mathrm{Var}\left(W\cdot g_{\mathrm{sg}}\right) & =W\cdot G_{{\mathrm{CAG}}_{\mathrm{sg}}}\cdot W^{\prime }\cdot \sigma_{a_{\mathrm{sg}}}^{2}\\ & =G_{{\mathrm{CAG}}_{\mathrm{sg}}}\cdot \sigma_{a_{\mathrm{sg}}}^{2}\\ & =\frac{Z_{\mathrm{sg}}\cdot R_{\mathrm{sg}}\cdot {Z^{\prime }}_{\mathrm{sg}}}{s_{\mathrm{sg}}}\cdot \sigma_{a_{\mathrm{sg}}}^{2}\\ & =Z_{\mathrm{sg}}\cdot R_{\mathrm{sg}}\cdot {Z^{\prime }}_{\mathrm{sg}}\cdot \sigma_{m_{\mathrm{sg}}}^{2}\\ & =\mathrm{Var}\left(Z_{\mathrm{sg}}\cdot m_{\mathrm{sg}}\right). \end{array}$$

This proof demonstrates that the variance of the GASC-BLUP model is identical to its SNP-BLUP equivalent, ensuring the models' theoretical consistency.
