# Supplementary material for: Genomic prediction in a backcross population using relationship matrices
Source: Arch Anim Breed. 2025 Jun 17;68(2):377–94. doi: 10.5194/aab-68-377-2025 (PMC13283310; doi:10.5194/aab-68-377-2025)
Supplement: The supplement related to this article is available online at https://doi.org/10.5194/aab-68-377-2025-supplement. [file aab-68-377-2025-supplement.pdf]

Supplement of Arch. Anim. Breed., 68, 377–394, 2025
https://doi.org/10.5194/aab-68-377-2025-supplement
© Author(s) 2025. CC BY 4.0 License.

Open Access
Archives Animal Breeding

Supplement of

Genomic prediction in a backcross population using relationship matrices

Abdulraheem A. Musa et al.

Correspondence to: Abdulraheem A. Musa (musa@fbn-dummerstorf.de)

The copyright of individual parts of the supplement might differ from the article licence.

Supplementary Table S1. Mean performance of the models in various scenarios of heritabilities and marker densities in simulations of backcross populations

Simulated				Model	Estimated parameters				
h^2	MD	σ_g^2	σ_e^2		$\hat{\sigma}_a^2$ (rMSE)	$\sigma_{\hat{g}}^2$ (rMSE)	$\hat{\sigma}_e^2$ (rMSE)	Accuracy ($r_{\hat{g},g}$)	rMSE of GEBV
**Independent simulation**									
220	0.17	3.27	16	G-BLUP	3.19(0.89)	1.78(1.62)	16.09(1.12)	0.76	1.2
				CAG-BLUP	4.3(1.78)	1.7(1.69)	16.77(1.33)	0.74	1.23
				GASI-BLUP	3.04(1.01)	2.11(1.34)	16.14(1.12)	0.78	1.14
				GASC-BLUP	6.14(5.46)	2.01(1.43)	16.67(1.28)	0.77	1.18
	0.7	3.27	1.4	G-BLUP	2.79(0.56)	2.67(0.66)	1.53(0.18)	0.93	0.65
				CAG-BLUP	8.53(5.57)	2.59(0.74)	1.75(0.38)	0.92	0.72
				GASI-BLUP	2.66(0.68)	2.79(0.55)	1.54(0.19)	0.94	0.6
				GASC-BLUP	19.66(17.8)	2.75(0.59)	1.52(0.21)	0.94	0.63
440	0.17	3.27	16	G-BLUP	3.27(0.91)	1.81(1.59)	16.03(1.11)	0.77	1.18
				CAG-BLUP	4.33(1.84)	1.72(1.68)	16.78(1.33)	0.75	1.22
				GASI-BLUP	3.15(1.03)	2.13(1.33)	16.08(1.11)	0.79	1.13
				GASC-BLUP	6.38(5.69)	2.02(1.42)	16.67(1.28)	0.77	1.18
	0.7	3.27	1.4	G-BLUP	3.04(0.39)	2.72(0.61)	1.43(0.12)	0.94	0.62
				CAG-BLUP	9.31(6.36)	2.62(0.71)	1.73(0.35)	0.92	0.7
				GASI-BLUP	2.94(0.49)	2.85(0.5)	1.44(0.12)	0.95	0.57
				GASC-BLUP	24.18(23.05)	2.78(0.56)	1.45(0.2)	0.94	0.61
**Dependent simulation**									
220	0.17	6.54	31.93	G-BLUP	6.47(1.29)	4.2(2.57)	31.3(2.24)	0.81	1.51
				CAG-BLUP	8.97(3.05)	4.39(2.39)	32.31(2.13)	0.83	1.43
				GASI-BLUP	4.78(2.26)	5.66(1.49)	31.74(2.09)	0.92	1.04
				GASC-BLUP	12.51(14.72)	5.6(1.49)	32.17(2.09)	0.92	1.05
	0.7	6.54	2.8	G-BLUP	4.39(2.18)	5.73(0.91)	2.78(0.22)	0.95	0.79
				CAG-BLUP	9.26(3.1)	5.66(0.96)	3.16(0.43)	0.95	0.84
				GASI-BLUP	3.11(3.45)	6.14(0.6)	2.92(0.24)	0.97	0.6
				GASC-BLUP	28.55(23.75)	6.15(0.59)	2.72(0.25)	0.97	0.59
440	0.17	6.54	31.93	G-BLUP	6.57(1.33)	4.24(2.53)	31.21(2.25)	0.82	1.49
				CAG-BLUP	9(3.07)	4.41(2.38)	32.34(2.13)	0.84	1.42
				GASI-BLUP	4.99(2.18)	5.69(1.48)	31.63(2.1)	0.92	1.01
				GASC-BLUP	12.72(13.83)	5.61(1.49)	32.19(2.1)	0.92	1.03
	0.7	6.54	2.8	G-BLUP	4.61(1.97)	5.79(0.86)	2.66(0.25)	0.95	0.77
				CAG-BLUP	9.47(3.29)	5.69(0.94)	3.16(0.42)	0.95	0.82
				GASI-BLUP	3.26(3.32)	6.2(0.57)	2.81(0.2)	0.98	0.55
				GASC-BLUP	36.11(32.04)	6.19(0.57)	2.62(0.31)	0.98	0.57

σ_g^2 : true genetic (Mendelian sampling) variance; σ_e^2 : true residual variance; $\hat{\sigma}_a^2$ and $\hat{\sigma}_e^2$: mean estimated additive and residual variance components, respectively; $\sigma_{\hat{g}}^2$: mean estimated genetic (Mendelian sampling) variance; rMSE: root mean squared error, indicating the deviation of estimated variances from true values; h^2 : heritability; MD: marker density; GEBV: genomic estimated breeding values; Accuracy ($r_{\hat{g},g}$): correlation between GEBV and true breeding values, indicating the precision of GEBV in predicting true breeding values.

Supplementary Table S2. Power (%) to detect a QTL-carrying chromosome in independent and dependent simulations of backcross populations

		h^2 = 0.17		h^2 = 0.29		h^2 = 0.70	
Chr	QE	G-BLUP	CAG-BLUP	G-BLUP	CAG-BLUP	G-BLUP	CAG-BLUP
**Independent simulation**							
1	0.5	5	5	29	27.5	89.5	89.5
2	1.0	88	86	99.5	99.5	100	100
3	0.25	0.5	1	2	2.5	7.5	7
4	0.50	5	4	19.5	15	86	79.5
5	0.50	8.5	6	22.5	20	83	78.5
6	0.1	-	-	-	-	-	-
7	0.25	1.5	1	1.5	1.5	11	12
8	0.25	1.5	1	1.5	1.5	7.5	8
9	-0.25	1.5	1	3	3	11.5	13.5
10	0.5	7.5	8	29.5	29.5	90.5	86.5
11	1.0	83.5	82	99	99	100	100
12	0.1	-	-	-	-	0.5	0.5
16	-	0	0.5	-	0.5	-	0.5
**Dependent simulation**							
1	1.0, 1.0, 0.5	100	100	100	100	100	100
2	0.5, 0.5, 0.25, 0.5	93	91.5	100	100	100	100
3	0.1, 0.25, 0.1	1	1	2	3	20	19.5
4	-0.25, 0.25	-	-	-	-	0.5	0.5
5	-	-	-	-	-	0.5	0.5
11	-	0	0.5	0	0.5	-	-
12	-	0.5	0.5	0.5	0.5	0.5	0.5
14	-	0.5	0	0.5	-	-	-
16	-	0	0.5	0.5	0.5	-	-
17	-	-	-	-	-	0.5	0.5
19	-	0.5	0.5	0.5	0.5	0.5	0.5

h^2 = heritability; QE = simulated QTL effect size; Chr = chromosome.
Results presented are for scenarios with a marker density of 2020 markers and chromosomes with entries.

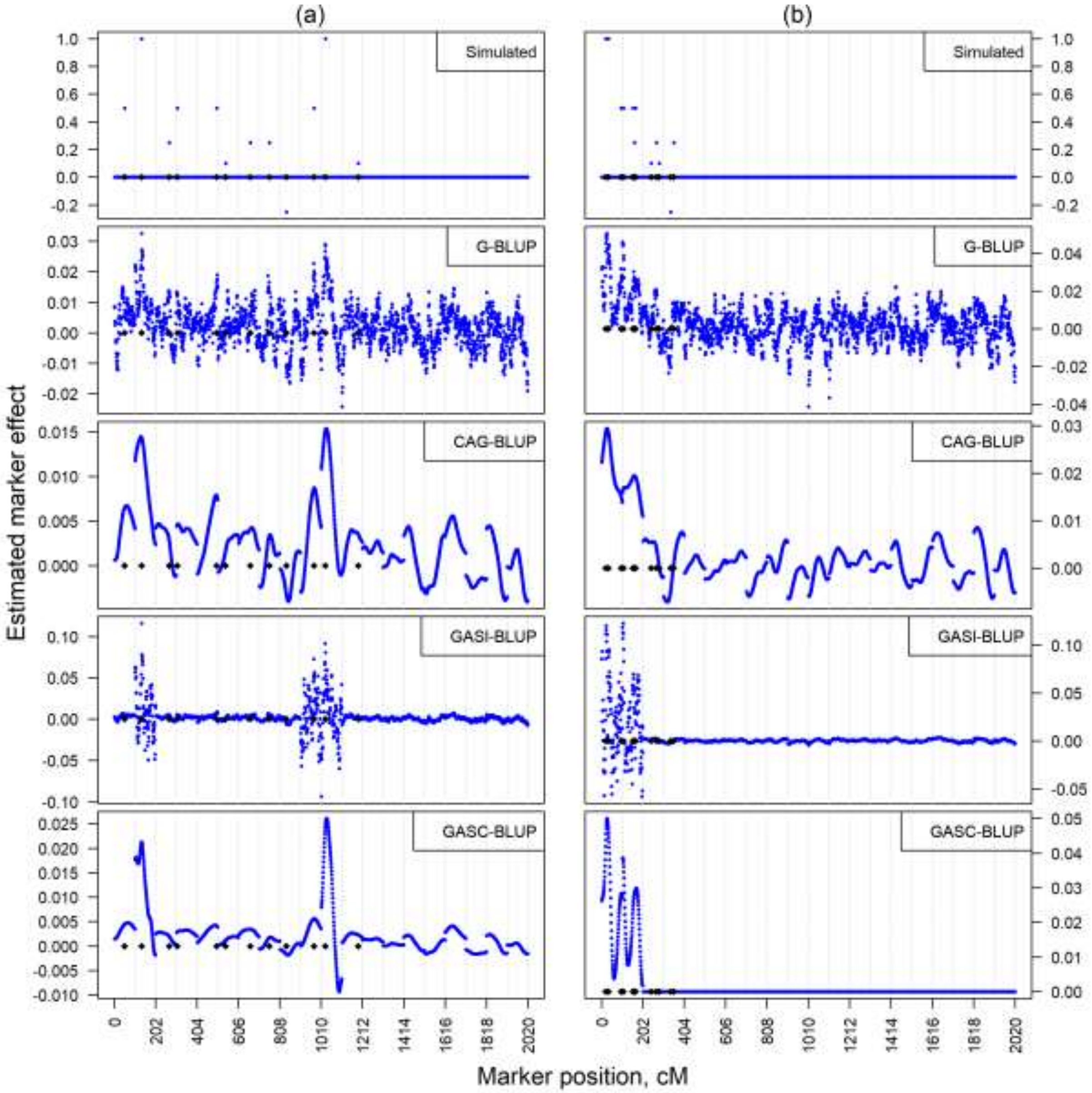

15 Supplementary Figure S1. Simulated and estimated marker effects of a randomly selected replicate in independent (panel a) and dependent (panel b) simulations with a 1 cM marker distance and heritability of 0.29. The black diamonds are the quantitative trait loci positions.
